# UDP-glucosyltransferase PpUGT85A2 controls volatile glycosylation in peach

**DOI:** 10.1093/jxb/ery419

**Published:** 2018-11-27

**Authors:** Boping Wu, Xiangmei Cao, Hongru Liu, Changqing Zhu, Harry Klee, Bo Zhang, Kunsong Chen

**Affiliations:** 1 Laboratory of Fruit Quality Biology/Zhejiang Provincial Key Laboratory of Horticultural Plant Integrative Biology, Zhejiang University, Zijingang Campus, Hangzhou, PR China; 2 Horticultural Sciences, Plant Innovation Center, Genetic Institute, University of Florida, Gainesville, FL, USA

**Keywords:** Flavor, fruit, defense, volatile, RNA-Seq, UGT

## Abstract

The monoterpene linalool is a major contributor to aroma and flavor in peach (*Prunus persica*) fruit. It accumulates during fruit ripening, where up to ~40% of the compound is present in a non-volatile glycosylated form, which affects flavor quality and consumer perception by retronasal perception during tasting. Despite the importance of this sequestration to flavor, the UDP-glycosyltransferase (UGT) responsible for linalool glycosylation has not been identified in peach. *UGT* gene expression during peach fruit ripening and among different peach cultivars was analyzed using RNA sequencing, and transcripts correlated with linalyl-β-d-glucoside were selected as candidates for functional analysis. Kinetic resolution of a racemic mixture of *R*,*S*-linalool was shown for PpUGT85A2, with a slight preference for *S*-(+)-linalool. PpUGT85A2 was shown to catalyze synthesis of linalyl-β-d-glucoside *in vitro*, although it did not exhibit the highest enzyme activity between tested substrates. Subcellular localization of PpUGT85A2 in the cytoplasm and nucleus was detected. Application of linalool to peach leaf disks promoted *PpUGT85A2* expression and linalyl-β-d-glucoside generation. Transient expression in peach fruit and stable overexpression in tobacco and Arabidopsis resulted in significant accumulation of linalyl-β-d-glucoside *in vivo*. Taken together, the results indicate that *PpUGT85A2* expression is a major control point predicting linalyl-β-d-glucoside content.

## Teaser

Synthesis of the major glycosylated monoterpene linalool in peach is catalyzed by PpUGT85A2.

## Introduction

Plants produce and release volatile chemicals during growth and development. These volatiles have important organoleptic properties in the edible portions of plants, including fruit, contributing to flavor and impacting consumers’ preferences. As an economically important fruit, peach is a model for the Rosaceae family (Shulaev *et al.*, 2008), whose volatiles have been intensively studied. More than 100 compounds have been identified in peach fruit, including aldehydes, alcohols, esters, lactones, and terpenes (Zhang *et al.*, 2010). Among these volatiles, ~20 compounds have been proposed to impact flavor (Eduardo *et al.*, 2010; Bianchi *et al.*, 2017; Tian *et al.*, 2017). The acyclic monoterpene linalool, imparting a sweet, floral, and alcoholic note, is a major contributor to the aroma of peach fruit (Tian *et al.*, 2017). A recent study revealed that UV-B irradiation reduced linalool production, impaired consumers’ liking of the aroma, and identified a terpene synthase gene (*PpTPS1*) responsible for its formation (Liu *et al.*, 2017).

In addition to the contribution to aroma and flavor quality, linalool is also considered to function in plant defense. Neural and behavioral sensitivity to linalool emitted by Arabidopsis was observed for fig wasps, fungus gnats, and moths (Raguso, 2016). Linalool has been reported to confer resistance to rice bacterial blight caused by *Xanthomonas oryzae* pv. *oryzae* (*Xoo*) (Taniguchi *et al.*, 2014). In sweet orange, transgenic overexpression of a linalool synthase gene, *CuSTS3-1*, led to significantly higher content of linalool and strong resistance to citrus canker induced by *Xanthomonas citri* subsp. *citri* (*Xcc*) (Shimada *et al.*, 2017). Antibacterial and antifungal activities of linalool against *Xcc* and *Penicillium italicum* were observed in Ponkan mandarin (*Citrus reticulata* Blanco) accumulating a high level of linalool in leaves and fruit peel (Shimada *et al.*, 2014). Moreover, a cytotoxic effect of linalool induced mitochondrial ROS (reactive oxygen species) production and apoptotic cell death (Gu *et al.*, 2010; Cheng *et al.*, 2017). All of these results are consistent with a defensive function of linalool in plants.

Plant secondary metabolites are frequently glycosylated. Volatile compounds may be present in fruits as both free and glycosidically bound forms. The free volatiles can be quickly released from fruits, while the conjugated volatiles are non-volatile molecules. These odorless conjugated volatiles can be liberated through acid or enzymatic hydrolysis during fruit ripening or upon tissue damage, potentially affecting fruit aroma and defense responses. Increased floral and peach-like aroma was observed for peach juice after enzymatic hydrolysis by β-glucosidase, accompanying a higher content of linalool (Tian *et al.*, 2017). Glycosylated linalool has been detected in grape (Bönisch *et al.*, 2014), kiwifruit (Yauk *et al.*, 2014), mandarin (Fan *et al.*, 2009), and peach fruit (Wu *et al.*, 2017). Glycosylation reactions are catalyzed by UDP-glycosyltransferases (UGTs), which transfer sugar molecules to acceptor aglycones. For example, a UGT attaches a glucose moiety to the hydroxyl position of linalool to form linalyl-β-d-glucoside (Bönisch *et al.*, 2014). The plant UGT family is quite large. Some plant UGTs have been characterized, including enzymes associated with metabolism of phytohormones (Yonekura-Sakakibara and Hanada, 2011), anthocyanins (Cheng *et al.*, 2014), flavonols (Ono *et al.*, 2010), and phloretin (Dare *et al.*, 2017). Although a few UGT genes responsible for the formation of glycosylated volatiles have been characterized in tomato (Tikunov *et al.*, 2013), grape (Bönisch *et al.*, 2014), kiwifruit (Yauk *et al.*, 2014), and strawberry (Song *et al.*, 2016), little is known about volatile glycosylation in peach fruit. Our recent study characterized the UGT gene family in peach fruit, revealing expression patterns, and detecting changes in glycosylated linalool during fruit ripening (Wu *et al.*, 2017). These results prompted us to identify further those UGT genes that are associated with formation of glycosylated linalool. Understanding the expression of the responsible UGT-encoding gene is an important step in controlling the pool of this chemical for enhanced aroma and defense in peach.

In this study, we first screened candidate *UGT* genes for correlations between transcript levels and content of linalyl-β-d-glucoside during peach fruit ripening, in response to ethylene treatment, between peach tissues and among different peach cultivars. Using the peach genome database and phylogenetic analysis, UGT genes with potential function in production of linalyl-β-d-glucoside were cloned. *In vitro* enzymatic assays indicated that PpUGT85A2 catalyzes linalool glycosylation. Application of linalool to peach leaf tissue induced accumulation of *PpUGT85A2* transcripts and linalyl-β-d-glucoside. Transient expression in peach fruit and stably transformed Arabidopsis and tobacco plants was used to demonstrate that *PpUGT85A2* synthesizes glycosylated linalool *in planta*. Together, the data support a model in which *PpUGT85A2* controls glycosylation of linalool.

## Materials and methods

### Plant materials

Peach (*Prunus persica* L. Batsch cv. Hujingmilu) fruit, flowers, and leaves were obtained from the Melting Peach Research Institute of Fenghua, Zhejiang Province, China in 2015. Peach fruit were harvested at five developmental stages, representing the first fast growth (34 days after bloom, DAB), endocarp lignification (stone hardening, 71 DAB), the second fast growth (94 DAB), and the mature stage (108 DAB and 111 DAB) (Wu *et al.*, 2017). Peach fruit harvested at 94 DAB were divided into several batches for treatment. To accelerate ripening, the first batch was treated with 100 μl l^−1^ ethylene for 12 h at 20 °C. To delay fruit ripening, peach fruit were treated with the ethylene signaling inhibitor *1-methylcyclopropene* (1-MCP) at 5 μl l^−1^ for 12 h at 20 °C in a sealed 20 liter container. Control fruit were incubated in an atmosphere without added ethylene or 1-MCP. Fans were used in the container to maintain air circulation. After treatment for 3 d, ethylene production was measured and fruit were sampled. To investigate the effects of defense signal on linalool production, peach fruit were treated with 1 mM salicylic acid (SA). After immersion at 20 °C for 5 min, the fruit were air dried and then stored at 20 °C for 12 h. For UV-B treatment, peach fruit were exposed to irradiation at 1.5 w m^–2^ for 48 h at 20 °C according to Wu *et al.* (2017). To investigate the distribution of linalyl-β-d-glucoside in cultivars, fruit of eight peach cultivars (*Prunus persica*) were sampled at the ripening stage, namely ‘Hanlumi’ (HLM), ‘Dahe’ (DH), ‘Baifeng’ (BF), ‘Zaoshanghaishuimi’ (ZSHSM), ‘Fuyangchiyue’ (FYCY), ‘Hujingmilu’ (HJML), ‘Hongpan2’ (HP2), and ‘Hongpan1’ (HP1). Three biological replicates, each with five fruit, were collected and immediately frozen in liquid nitrogen, then stored at –80 °C until further analysis.

### Extraction and hydrolysis of glycosylated volatiles for GC-MS

Glycosylated volatiles were extracted according to Wu *et al.* (2017). Frozen peach fruit (20 g), leaf (5 g), flower (5 g), tobacco leaf (1 g), and Arabidopsis plants (1 g) were ground and homogenized with water. After centrifuging for 20 min at 13 000 *g*, the supernatant was used as the crude extract. Isolation of glycosidic precursors was conducted by using SPE LC-18 resins (CNW, Duesseldorf, Germany). Elimination of free volatile compounds was performed by washing with 25 ml of dichloromethane, and the bound fraction was eluted with 25 ml of methanol. The bound volatile compounds were enzymatically hydrolyzed at 37 °C after adding 2 mg of AR2000 (Rapidase, Séclin, France) according to previous studies (Bönisch *et al.*, 2014; Yauk *et al.*, 2014; Gao *et al.*, 2018). The free aglycones were released for 30 min at 45 °C and collected using a solid-phase microextraction (SPME) fiber coated with 65 μm of polydimethylsiloxane and divinylbenzene (PDMS–DVB) (Supelco Inc., Bellefonte, PA, USA). The released volatiles were identified using GC-MS.

An Agilent 7890N gas chromatograph coupled with an Agilent 5975C mass spectrophotometer (Agilent, Palo Alto, CA, USA) equipped with a DB-WAX column (0.32 mm, 30 m, 0.25 μm, J&W Scientific, Folsom, CA, USA) was applied for identification of volatile compounds according to methods described by Liu *et al.* (2017). Helium was used as a carrier gas at a flow rate of 1.0 ml min^−1^. The temperature program started at 40 °C and was increased by 3 °C min^−1^ to 100 °C and then to 245 °C at 5 °C min^−1^. The column effluent was ionized by electron ionization (EI) at an energy of 70 eV with the transfer temperature of 250 °C, and the source temperature of 230 °C. Mass scanning was done over the range 35–350 *m/z*. Volatile compounds were identified by comparing their EI mass spectra with the NIST/EPA/NIH Mass Spectral Library (NIST-08) and the retention time of authentic standards.

### Free volatile analysis by GC-MS

Free volatiles were analyzed according to Zhang *et al.* (2010). Frozen peach fruit (5 g), leaf (1 g), flower (1 g), tobacco leaf (1 g), and Arabidopsis plants (1 g) were ground and transferred to vials containing a solution of 200 mM EDTA and 20% CaCl_2_. 2-Octanol (0.7 mg ml^−1^) was used as an internal standard. Free volatiles were extracted using fibers coated with 65 μm of PDMS–DVB (Supelco Co.) for 30 min at 45 °C, and identified using GC-MS as mentioned above.

### Chiral GC-MS analysis

The enantiomers of linalool were separated using a 7890N gas chromatograph coupled to an Agilent 5975C mass spectrometer equipped with a β-Dex™ 325 capillary column (30 m×0.25 mm×0.25 μm; Supelco Co.) according to methods described by Liu *et al.* (2017). Helium was used as carrier gas. The temperature program was as follows: initial temperature 35 °C increased to 118 °C by 5 °C min^−1^, then increased to 118.4 °C, followed by a further increase to 230 °C by 5 °C min^−1^. Authentic standards were used for identification of linalool enantiomers.

### HPLC and LC/triple quadrupole (TQ) MS analysis

The glucosides produced by the recombinant proteins were dissolved in methanol and analyzed by the Agilent 1290 Infinity LC System (binary pump G4220A, diode array UV/VIS detector G4212A; Agilent Technologies) coupled with a SunFire C18 analytical column (5 μm, 4.6 × 250 mm; Waters, USA). The chromatograms were obtained and detected between 200 nm and 400 nm. HPLC were performed at a flow rate of 1 ml min^−1^ with 100% water as solvent A and 100% acetonitrile as solvent B. The injection volume of samples was 20 ml. The column was first equilibrated with 10% solvent B and eluted with a linear gradient program from 30% to 70% solvent B for 15 min, then washed with 100% solvent A for 15 min. The MS analyses were performed with an Agilent6460 triple quadrupole mass spectrometer equipped with negative ion electrospray ionization (ESI) MS in full scan mode. The scan range was 100–1000 *m/z*, and the nebulizer pressure was set as 45 psi. Identification of glucoside was based on the HPLC retention time and MS spectral data. Linalyl-β-d-glucoside was quantified by absorption at 210 nm, and the *m/z* value was 315 [M-H]^−^ according to Bönisch *et al.* (2014).

### Heterologous protein expression and purification

Full-length ORFs of the peach UGT gene were cloned into the pET6xHN expression vector (Clontech, Palo, CA, USA) using the primers listed in Supplementary Table S5 at *JXB* online. After sequencing, recombinant proteins were expressed in *Escherichia coli* BL21 (DE3) pLysS (Promega, Madison, WI, USA) according to Wu *et al.* (2017). The protein expression was induced with 1.0 mM isopropyl-β-d-thiogalactopyranoside (IPTG). The cells were harvested by centrifugation (4000 *g*, 4 °C, 15 min) after incubation at 16 °C at 150 rpm for 20 h. After storing at –80 °C overnight, the cells were obtained by centrifugation (10 000 *g*, 4 °C, 30 min), and then purified using a TALON Spin column (Clontech). The purified protein was obtained by linear gradient elution and confirmed by SDS–PAGE.

### Enzymatic activity assay

Enzymatic activity assays were carried out according to Wu *et al.* (2017). Reactions were performed in 200 μl of reaction mixture, containing 100 mM Tris–HCl buffer (pH 7.5, and 2.0 mM DTT), 1.0 mM UDP-sugar (UDP-glucose or UDP-galactose), 1.0 mM substrates, and purified protein (0.4–0.5 μg μl^−1^). After incubation at 30 °C for 16 h, the reaction was terminated by addition of 1 ml of 24% (v/v) trichloroacetic acid (TCA), extracted with ethyl acetate, and then evaporated to dryness. The glucosides were dissolved in methanol and analyzed by LC/TQMS. The optimal reaction temperature was determined in the range of 20–50 °C at pH 7.5. The optimal pH was determined in the range of pH 5.5–8.5. To determine the kinetic parameters of the acceptor, the concentration of substrate ranged from 5 μM to 20 mM, with UDP-glucose at 1 mM. To analyze the kinetic parameters of the sugar donor, 1 mM linalool was used as acceptor and the concentration of UDP-glucose ranged from 25 μM to 5 mM. All the kinetic assays were incubated at 30 °C for 1 h and repeated in triplicate. The kinetic parameters *K*_m_ and *V*_max_ were calculated from Lineweaver–Burk plots and Hanes–Woolf plots.

### Peach disk experiment

Peach (*P. persica* L. Batsch cv. Hujingmilu) leaves were used to investigate the effects of linalool on expression of *UGTs* and the content of glycosylated linalool according to Zhang *et al.* (2009) with modifications. Leaf disks were drilled using a 8 mm diameter cork borer, briefly rinsed with sterile water, and collected in 0.4 M mannitol. For each treatment, four replicates of 16 g leaf disks were placed into 250 ml conical flasks containing 200 ml of the treatment solutions. Leaf disks were treated with 1.0 mM linalool in 0.4 M mannitol containing 0.01% (v/v) Triton X-100, and the same solution without linalool as the negative control. The experiment was performed by shaking the disks for 24 h at 100 rpm at 28 °C. After 24 h incubation, the disks were blotted dry on filter paper, frozen in liquid nitrogen, and stored at –80 °C until use for chemical content and gene expression analysis.

### Phylogenetic tree construction

A phylogenetic tree was constructed with the FigTree v1.4.2 program, by aligning the full-length amino acid sequences of UGTs using the Neighbor–Joining (NJ) method in the ClustalX v2.0 program. Sequences used for phylogenetic tree analysis include *Vitis vinifera* VvGT14 (XM_002285734.2), *Actinidia deliciosa* AdGT4 (KF954944), *Fragaria×ananassa* FaUGT71W2 (XP_011468178.1), FaUGT73B23 (XP 004304022.1), and FaUGT73B24 (XP 004304022.1). Ten UGTs of *Arabidopsis thaliana*, namely UGT73C1, UGT73C5, UGT73C6, UGT75B1, UGT75B2, UGT75D1, UGT76D1, UGT76E11, UGT84B1, and UGT84B2 were used. The peach UGT sequences were obtained from the peach genome database Phytozome (v11.0, https://phytozome.jgi.doe.gov/) according to Wu *et al.* (2017).

### Gene expression analysis

Total peach RNA was extracted according to the protocol described by Zhang *et al.* (2006). For tobacco and Arabidopsis plants, total RNA was isolated using the TRIzol Reagent (Ambion, Hopkinton, MA, USA) kit. Quantitative real-time PCR gene expression analysis was performed on a CFX96 instrument (Bio-Rad, Hercules, CA, USA) using Ssofast EvaGreen Supermix (Bio-Rad). Primers are described in Supplementary Table S5. RNA sequencing (RNA-Seq) was performed on an Illumina HiSeq 2500 sequence platform and data were analyzed as described previously (Zhang *et al.*, 2016). Transcript abundance was expressed as RPKM (reads per kilobase of exon model per million mapped reads) based on the length of the gene and the number of reads mapped to this gene. The resulting *P*-values were adjusted to control the FDR (false discovery rate). Three biological replicates were performed for RNA isolation and gene expression analysis.

### Gene transient overexpression in peach fruit

Full-length *PpUGT85A2* was amplified and cloned into the pGreen II 0029 62-SK vector (EU048865), using the primers listed in Supplementary Table S5. The recombined vector was transformed into *Agrobacterium tumefaciens* strain GV3101::pSoup by electroporation. Transient overexpression in non-melting peach (*P. persica* L. Batsch cv. Hanlumi) fruit was performed according to Liu *et al.* (2017). The constructs containing *PpUGT85A2* and empty vector in *Agrobacterium* were infiltrated into the same fruit on opposite sides of the flesh cubes (1 cm thick) under a –70 kPa vacuum. After vacuum infiltration, flesh cubes were rinsed three times with sterile water, and cultured on Murashige and Skoog (MS) medium in a growth chamber (20 °C) for 3 d. Flesh cubes were then frozen in liquid nitrogen, and stored at –80 °C until further analysis. Transient expression treatments were repeated three times with five fruit in each replicate. The expression of *PpUGT85A2* was detected by qPCR analysis using the primers listed in Supplementary Table S5.

### Plant transformation and growth

Transgenic tobacco plants (*Nicotiana tabacum*) were regenerated as described by Wang *et al.* (2016) and rooted in the presence of kanamycin (150 mg l^−1^). Transgenic Arabidopsis plants were generated by *Agrobacterium* using the floral dip method and rooted in the presence of kanamycin (50 mg l^−1^). Plants were self-pollinated to produce homozygous lines. Transgenic and wild-type (WT) plants were grown in a greenhouse at 25 °C and 16 h light/8 h darkness. Three plants of each line were sampled for further analysis. For tobacco plants, full extended mature leaves were harvested. For Arabidopsis, the whole plants were harvested for analysis after 1 month of growth. To analyze glycosylated volatiles, pools of five tobacco leaves and three Arabidopsis plants were collected, frozen in liquid nitrogen, and stored at –80 °C.

### Subcellular localization analysis

The recombined 35S-*PpUGT85A2*-GFP vectors were constructed using primers listed in Supplementary Table S5, and were transformed into *A. tumefaciens* strain GV3101::pSoup by the Gene Pluser Xcell Electroporation System (Bio-Rad) according to Li *et al.* (2017). To identify the intracellular structure, the vector was infiltrated into transgenic tobacco (*Nicotiana benthamiana*) plants expressing a red fluorescent nuclear marker (Nucleus–RFP). After 3 d infiltration, the green fluorescent protein (GFP) fluorescence of tobacco leaves was imaged using a Zeiss LSM710NLO confocal laser scanning microscope. Three replications were performed to confirm subcellular localization.

### Statistical analysis

Figures were produced by Origin Pro 9.0 (Origin Lab Corporation, Northampton, MA, USA). MetaboAnalyst 3.0 (http://www.metaboanalyst.ca/) was used for correlation analysis between transcripts of UGT genes and contents of glycosylated linalool. The two-sample significance test was analyzed using two-tailed Student’s *t*-test, and Tukey HSD test was used to detect significant differences among groups at a significance level of 0.05 (SPSS 19.0, SPSS Inc., Chicago, IL, USA).

### Chemicals and reagents

Reference compounds used for identification of volatiles, including linalool, geraniol, eugenol, 2-phenylethanol, benzyl alcohol, and α-terpineol, were purchased from Sigma-Aldrich. Different sugar donors used for UGT enzyme activity analysis were supplied by Sigma-Aldrich when available, including UDP-glucose and UDP-galactose. 1-MCP was supplied by SmartFresh, and SA was purchased from Sigma-Aldrich. Silwet L-77, a surfactant for *Agrobacterium*-based transformations of Arabidopsis, was purchased from Real-Times (Beijing, China). Other reagents and solvents used in the present study were obtained from Shanghai Sangon Engineering and Biotechnology Services Co. Ltd.

## Results

### *PpUGT85A2* expression correlates with glycosylated linalool production in peach fruit

**Fig. 1.**
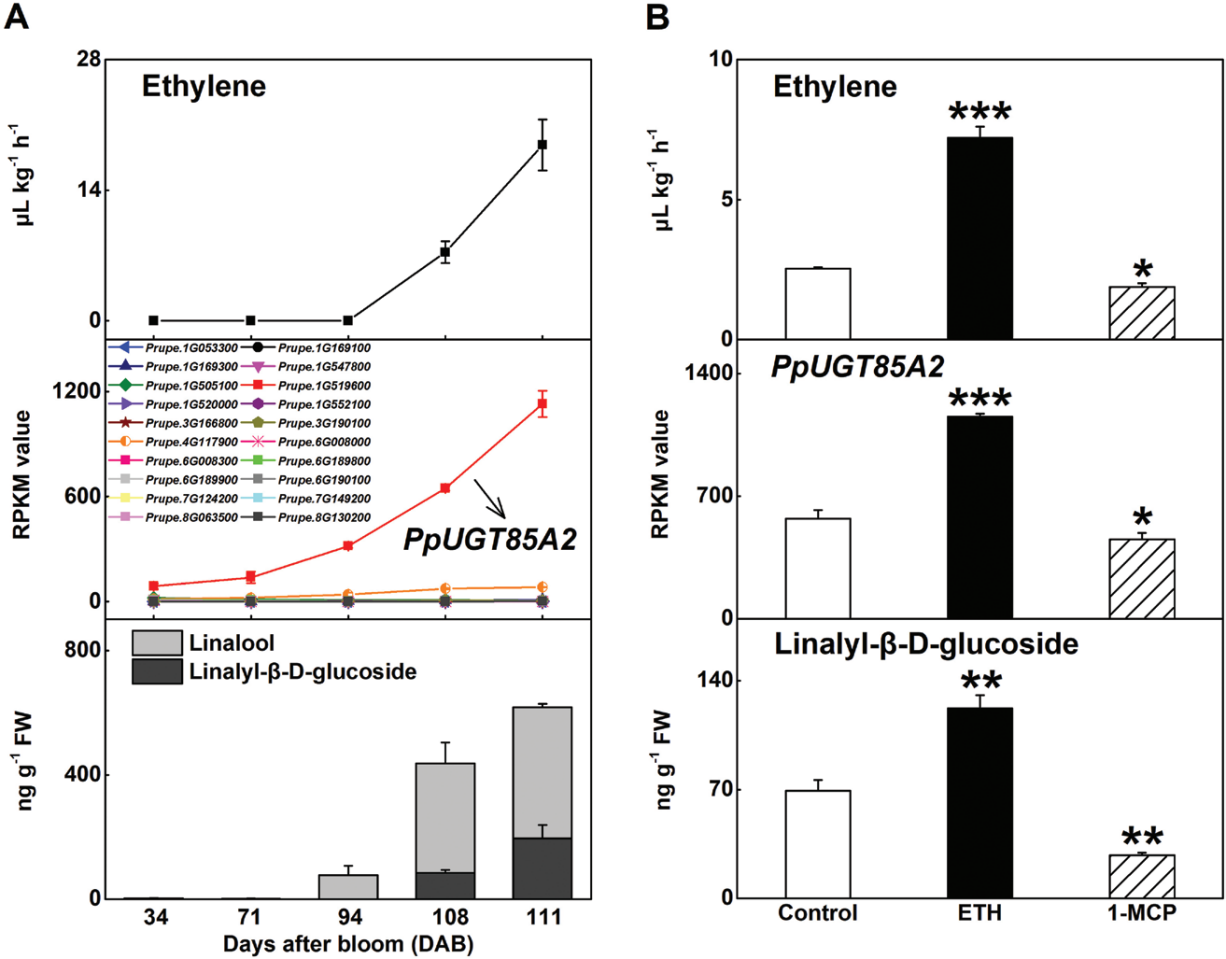
Expression of *PpUGT85A2* correlates with glycosylated linalool production in peach fruit. (A) Changes in ethylene production, transcript levels of 20 peach *UGT* genes, and content of linalyl-β-d-glucoside during fruit ripening. (B) Effect of ethylene and 1-MCP treatment on ethylene production, expression of *PpUGT85A2*, and content of linalyl-β-d-glucoside in peach fruit. Data are presented as the mean ±SE from three independent biological replicates. Significant differences are compared against controls and indicated with asterisks above the bars (*P<0.05, **P<0.01, ***P<0.001).

Linalool is a major monoterpene that contributes to flavor quality (Tian *et al.*, 2017), accounting for ~6% of total volatiles in peach fruit (Liu *et al.*, 2017). Approximately 34% of total linalool in ripe peach fruit harvested at 111 DAB (Fig. 1A) was present as non-volatile linalyl-β-d-glucoside. To identify peach *UGT* genes associated with the formation of linalyl-β-d-glucoside, a Pearson correlation analysis between *UGT* transcript levels and fruit glucoside contents was performed.

First, peach fruit at five different ripening stages were used for correlation analysis between *UGT* gene expression and linalyl-β-d-glucoside content (Wu *et al.*, 2017). A total of 20 *UGT* genes were selected for further analysis including the 10 *UGT* genes that had positive correlations and 10 *UGT* genes with negative correlations (Supplementary Table S1). The highest correlation coefficient was observed for *UGT Prupe.1G519600* (*PpUGT85A2*) (*R*=0.95, *P*<0.001). Following the correlation analysis, transcript levels of these *UGT* genes were investigated. Among these *UGT* genes, *PpUGT85A2* had the highest level of mRNA (Fig. 1A). During peach fruit ripening, transcript levels of *PpUGT85A2* correlated with increased production of linalyl-β-d-glucoside and ethylene production (Fig. 1A).

**Fig. 2.**
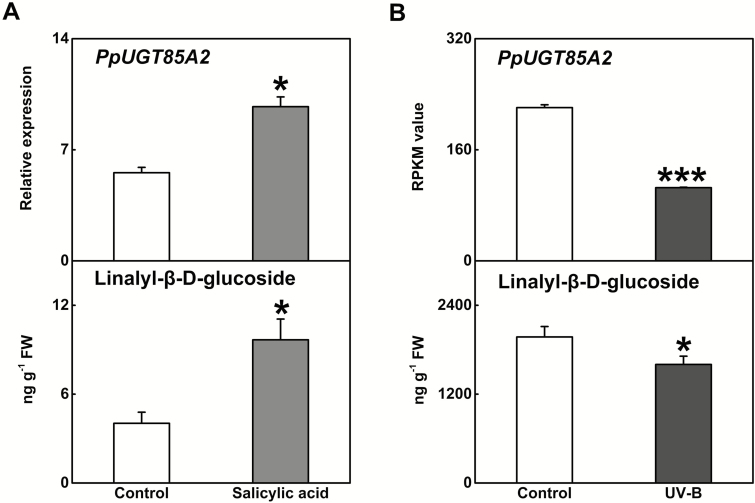
Effect of SA and UV-B treatment on content of glycosylated linalool and expression of *PpUGT85A2* in peach fruit. (A) Expression of *PpUGT85A2* and the content of linalyl-β-d-glucoside in peach fruit after SA treatment. (B) Changes in content of linalyl-β-d-glucoside and expression of *PpUGT85A2* following UV-B irradiation in peach fruit. Significant differences are indicated with asterisks above the bars (*P<0.05, ***P<0.001).

Ethylene regulates peach fruit ripening (Mathooko *et al.*, 2001). Treatment of fruit with exogenous ethylene stimulated internal ethylene synthesis as well as *PpUGT85A2* transcript and accumulation of linalyl-β-d-glucoside (Fig. 1B). In contrast, treatment of fruit with the ethylene signaling inhibitor 1-MCP reduced ethylene production and reduced both *PpUGT85A2* transcript and linalyl-β-d-glucoside content (Fig. 1B), and linalool content (Supplementary Fig. S1). For the 20 *UGT* genes mentioned above, *PpUGT85A2* had the highest transcript content in response to both ethylene and 1-MCP treatment (Supplementary Fig. S2), and positively correlated with linalyl-β-d-glucoside production (*R*=0.94, *P*<0.001) (Supplementary Table S2). SA treatment significantly increased *PpUGT85A2* transcript and the contents of linalyl-β-d-glucoside (Fig. 2A) as well as linalool (Supplementary Fig. S3). UV-B irradiation significantly reduced production of linalyl-β-d-glucoside and expression of *PpUGT85A2* in peach fruit (Fig. 2B), while transcripts of other *UGT* genes were induced in response to UV-B treatment (Supplementary Fig. S4; Wu *et al.*, 2017).

### Transcript levels of *PpUGT85A2* correlate with contents of linalyl-β-d-glucoside between peach organs and cultivars

**Fig. 3.**
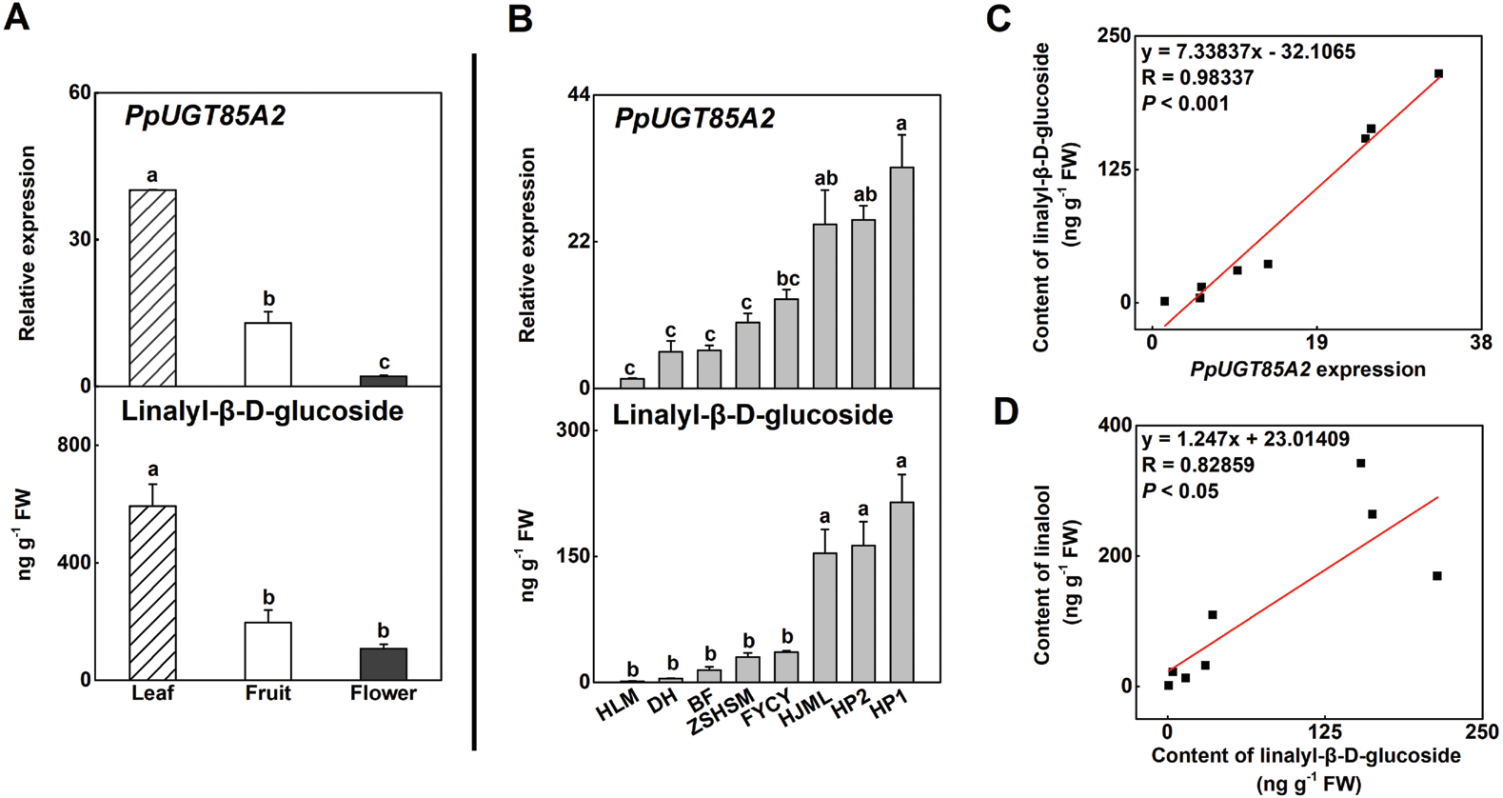
Expression of *PpUGT85A2* and content of linalyl-β-d-glucoside in peach. (A) Expression of *PpUGT85A2* and content of linalyl-β-d-glucoside in peach young leaf, open flower, and ripe fruit at 111 DAB. (B) Expression of *PpUGT85A2* and content of linalyl-β-d-glucoside in ripe fruit of eight peach cultivars. (C) Correlation between *PpUGT85A2* expression and linalyl-β-d-glucoside content in ripe fruit of eight peach cultivars. (D) Correlation between linalyl-β-d-glucoside and linalool content in ripe fruit of eight peach cultivars. Data are presented as the mean ±SE from three independent biological replicates. Values labeled with different letters indicate a significant difference at *P*<0.05.

To investigate the regulation of *PpUGT85A2* and linalyl-β-d-glucoside further, gene expression and chemical content were also determined in different organs and different peach cultivars. The highest content of linalyl-β-d-glucoside was detected in young leaf, followed by ripe fruit and fully open flower. Similar to the pattern of the glucoside, the highest transcript levels of *PpUGT85A2* were also observed in young leaf (Fig. 3A). To clarify the relationship between *PpUGT85A2* and linalyl-β-d-glucoside content further, we examined natural variation within eight different peach cultivars. Wide variations in linalyl-β-d-glucoside and *PpUGT85A2* transcript levels were observed among the different peach cultivars (Fig. 3B). Linear regression analysis showed that *PpUGT85A2* transcript contents strongly positively correlated with linalyl-β-d-glucoside content among the cultivars (*R*=0.98, *P*<0.001) (Fig. 3C). The content of linalyl-β-d-glucoside also correlated with free linalool (*R*=0.83, *P*<0.05) (Fig. 3D). Taken together, these results indicate a positive correlation between transcript and glucoside concentration, making *PpUGT85A2* a good candidate for linalyl-β-d-glucoside synthesis.

### Recombinant PpUGT85A2 protein catalyzes *in vitro* formation of linalyl-β-d-glucoside

**Fig. 4.**
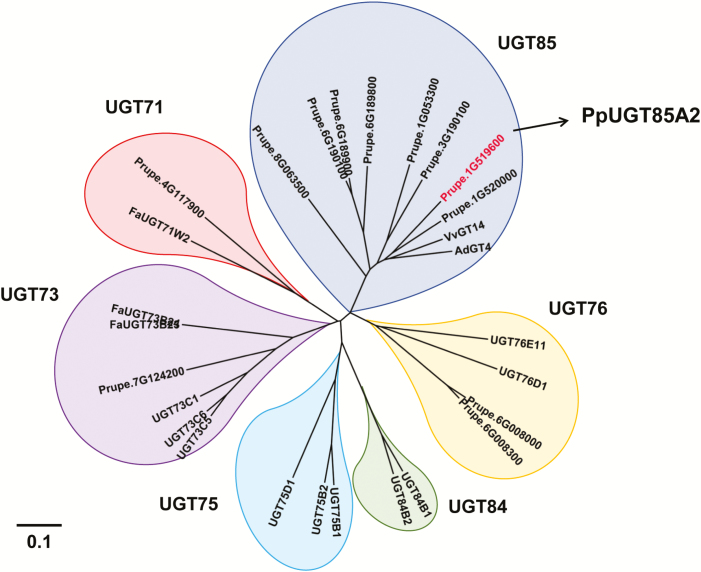
Phylogentic analysis of peach UGTs with other plant UGTs. *Vitis vinifera* VvGT14 (XM_002285734.2); *Actinidia deliciosa* AdGT4 (KF954944); and *Fragaria×ananassa* FaUGT71W2 (XP_011468178.1), FaUGT73B23 (XP 004304022.1), and FaUGT73B24 (XP 004304022.1). Ten UGTs of *Arabidopsis thaliana*, namely UGT73C1, UGT73C5, UGT73C6, UGT75B1, UGT75B2, UGT75D1, UGT76D1, UGT76E11, UGT84B1, and UGT84B2 were analyzed. The peach UGT sequences were obtained from the peach genome database Phytozome (v11.0, https://phytozome.jgi.doe.gov/).

*PpUGT85A2* encodes a 483 amino acid protein, belonging to the group G glycosyltransferase family based on phylogenetic analysis (Wu *et al.*, 2017). PpUGT85A2 is clustered with UGT85 family members that are associated with linalyl-β-d-glucoside formation (Fig. 4), including grapevine VvGT14 (Bönisch *et al.*, 2014) and kiwifruit AdGT4 (Yauk *et al.*, 2014), sharing 81% and 79 % amino acid similarity, respectively. In addition to the UGT85 family, gene products from the UGT71, UGT73, UGT75, UGT76, and UGT84 family have also been associated with linalool glycosylation (Caputi *et al.*, 2008; Song *et al.*, 2016). These results indicate that linalool can potentially be glycosylated by UGTs with diverse sequences and structures, indicating the difficulty of predicting function based on sequence homology and the importance of enzymatic characterization to identify functions of plant UGTs.

**Fig. 5.**
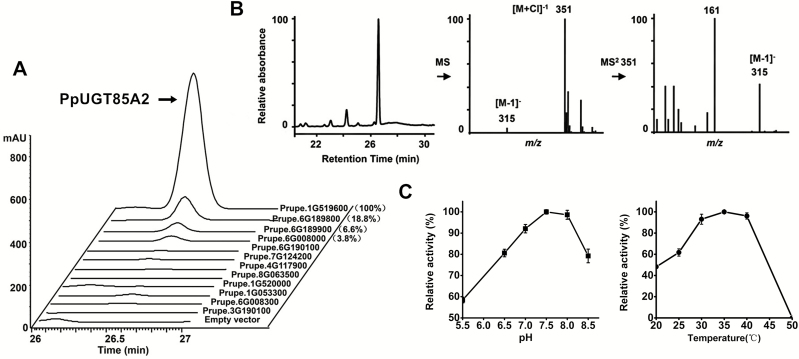
Enzymatic activity analysis of PpUGT85A2 protein. (A) Enzymatic activity of 12 recombinant peach UGT proteins toward linalool as substrate and UDP-glucose as sugar donor. The empty vector (pET6xHN) protein is shown as control. The retention time of linalyl-β-d-glucoside is ~26.5 min. UGT activity of PpUGT85A2 towards linalool is set at 100%. (B) LC/TQMS analysis of linalyl-β-d-glucosides formed by PpUGT85A2. Mass spectra of linalyl-β-d-glucoside consisted of a parent molecule ion [M-H]^−^ with mass/charge (*m/z*) 315 [315=154 (linalool)+162 (Glc-water)–H]^−^, and its chlorine adduct *m/z* 351 [351=315 + 36 (chlorine)]. (C) The optimum pH and temperature for PpUGT85A2 toward linalool and UDP-glucose.

To test further if the peach homologs could be associated with formation of linalyl-β-d-glucoside, a total of 12 *UGT* genes were cloned and expressed in *E. coli* (Fig. 5A). Enzymatic activity analysis showed that PpUGT85A2 (Supplementary Fig. S5) catalyzed formation of linalyl-β-d-glucoside (Fig. 5), with much higher activity than that of UGT85 family members Prupe.6G189800 (~18.8% activity in relative to PpUGT85A2) and Prupe.6G189900 (~6.6%) or the UGT76 family Prupe.6G008000 (~3.8%) (Fig. 5A). No linalool glucosylation activity was detected for the remaining eight UGTs, including UGT85 family Prupe.3G190100, Prupe.1G053300, Prupe.1G520000, Prupe.8G063500, and Prupe.6G190100, UGT71 family Prupe.4G117900, UGT73 family Prupe.7G124200, and UGT76 family Prupe.6G008300. These results indicate that while multiple peach UGT enzymes are capable of linalyl-β-d-glucoside synthesis, PpUGT85A2 had the highest *in vitro* activity. In addition, *PpUGT85A2* expression correlated positively (*R*=0.95) with the glycosylated linalool while *Prupe.6G189800* (*R*= –0.68), *Prupe.6G008000* (*R*= –0.43), and *Prupe.6G189900* (*R*= –0.33) were all negatively correlated with glycosylated linalool content (Supplementary Table S1). Based on all of these considerations, *PpUGT85A2* was selected for further biochemical and molecular analysis.

LC/TQMS analysis showed that recombinant PpUGT85A2 was able to catalyze *in vitro* formation of linalyl-β-d-glucoside using UDP-glucose as sugar donor (Fig. 5B), and had no activity toward linalool using UDP-galactose as sugar donor (Supplementary Fig. S6). The optimum pH for *in vitro* formation of linalyl-β-d-glucoside catalyzed by PpUGT85A2 was ~7.5–8.0, and the optimum temperature was in the range of 30–40 °C (Fig. 5C). Kinetic analysis showed that the *K*_m_ values for linalool and UDP-glucose were 0.463 mM and 0.684 mM, respectively. The specificity constant (*k*_cat_/*K*_m_) for linalool was 54 s^−1^ M^−1^ (Table 1). Different substrates were also tested for alcohols whose glycosylated forms were detected in peach fruit (Supplementary Fig. S7; Supplementary Table. S3). Geraniol was the best substrate among the monoterpenes, followed by linalool and α-terpineol. This relatively low activity for linalool was also observed with UGTs from grape (VvGT14a) (Bönisch *et al.*, 2014), kiwifruit (AdGT4) (Yauk *et al.*, 2014), and strawberry (FaUGT73B23, FaUGT73B24, and FaUGT71W2) (Song *et al.*, 2016).

**Table 1.** Analysis of the kinetic parameters of PpUGT85A2 recombinant protein

Substrate	*K*_m_ (mM)	*k*_cat_ (s^−1^)	*k*_cat_/*K*_m_ (s^−1^ M^−1^)
Linalool	0.463 ± 0.080	0.025 ± 0.005	54
UDP-glucose	0.684 ± 0.073	0.190 ± 0.025	278

**Fig. 6.**
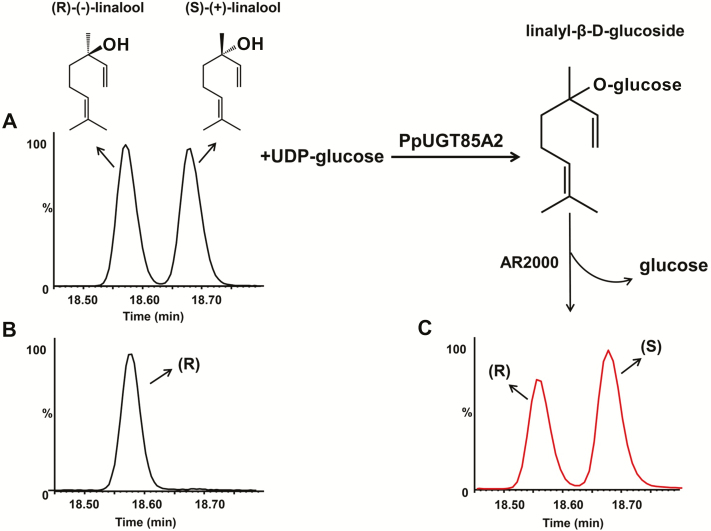
The enantiomeric preference of PpUGT85A2 towards linalool determined by chiral GC-MS analysis. (A) A racemic mixture (1:1) of *R*,*S*-linalool was used as a substrate. (B) Enantiomerically pure *R*-(–)-linalool was used as a reference material. (C) A slight enrichment of the *S*-(+)-linalool after hydrolysis of linalyl-β-d-glucoside by AR2000.

In ripe peach fruit, *S*-(+)-linalool is the major form of both free and glycosylated linalool (Liu *et al.*, 2017; Supplementary Fig. S8). To investigate the enantiomeric preference of PpUGT85A2, a racemic mixture (1:1) of *R*,*S*-linalool was used as a substrate, and enantiomerically pure *R*-linalool was used as reference material. Chiral-phase GC-MS analysis of linalyl-β-d-glucoside after hydrolysis by AR2000, showed a slight enrichment of *S*-(+)-linalool (Fig. 6). The enzyme AR2000 has no enatiomeric discrimination toward *R*,*S*-linalool (Bönisch *et al.*, 2014). Our data demonstrated low enantiomeric selectivity toward *R*-(–)-linalool during 16 h assays, different from results obtained with the grape VvGT14a enzyme (Bönisch *et al.*, 2014). Based on these results, the kinetic values for PpUGT85A2 were determined using a racemic mixture of linalool.

### Transient expression of *PpUGT85A2* in peach tissue

**Fig. 7.**
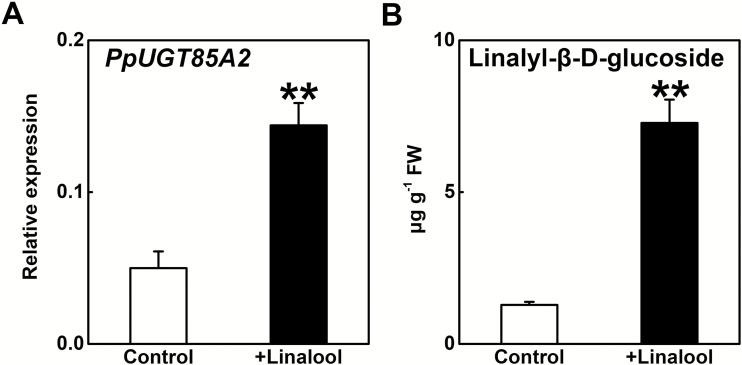
Effect of linalool treatment on expression of *PpUGT85A2* (A) and production of linalyl-β-d-glucoside (B) in peach disks. Solution without linalool was used as control. Data are presented as the mean ±SE from four independent biological replicates. Significant differences are indicated with asterisks above the bars (**P<0.01).

To investigate further the role of PpUGT85A2 in synthesis of linalyl-β-d-glucoside in peach, leaf disks were incubated with linalool. Transcript levels of *PpUGT85A2* were induced by ~3-fold after linalool treatment compared with the control (Fig. 7A). We also observed an ~5-fold increase in the content of linalyl-β-d-glucoside in these disks (Fig. 7B). These results indicated that *PpUGT85A2* expression in peach is induced by linalool, leading to production of linalyl-β-d-glucoside.

### Overexpression of *PpUGT85A2* increases production of linalyl-β-d-glucoside *in planta*

**Fig. 8.**
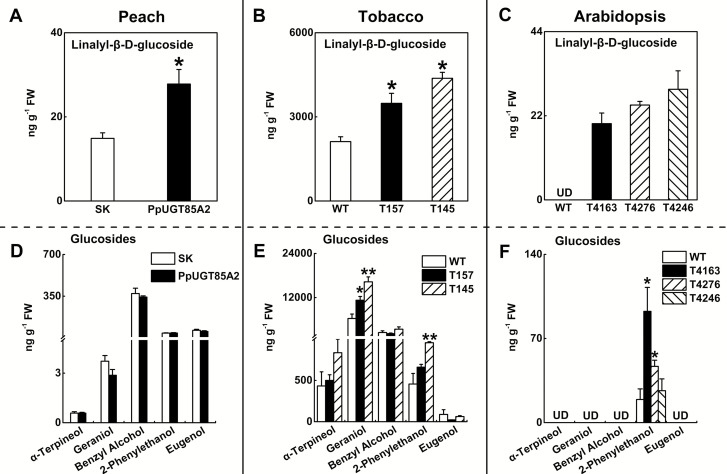
Changes in content of glucosides *in planta* after overexpressing *PpUGT85A2*. Linalyl-β-d-glucoside and other glucosides were isolated from peach fruit (A, D), from tobacco (B, E), and from Arabidopsis plants (C, F). Peach fruit were transiently overexpressing *PpUGT85A2*. Empty SK vector was used as a control. For tobacco and Arabidopsis transgenic plants, wild-type (WT) plants were used as controls. Data are presented as mean ±SE from three independent biological replicates. Significant differences are compared against empty SK vector or the WT, and indicated with asterisks above the bars (*P<0.05, **P<0.01). UD, under the limit of detection.

To our knowledge, production of transgenic peach fruits has not been reported. Recently, a transient expression system was developed in fruit for the purpose of gene function analysis (Liu *et al.*, 2017). Therefore, a homologous transient overexpression system combined with heterologous transgenic plant production was used to determine the function of *PpUGT85A2 in vivo*. There was a significant increase in *PpUGT85A2* transcript in fruit after infiltration with *Agrobacterium* harboring the pGreen-SK and PpUGT85A2 constructs (Supplementary Fig. S9). Introduction of the PpUGT85A2 vector resulted in an ~2-fold increase in linalyl-β-d-glucoside (Fig. 8A) and a corresponding depletion of free linalool (Supplementary Fig. S10A).

To test the function of the peach *UGT* gene further, *PpUGT85A2* was heterologously overexpressed in tobacco and Arabidopsis plants. Compared with WT plants, transgenic tobacco plants (T157 and T145) produced an ~1.5- and 2-fold higher content of linalyl-β-d-glucoside, respectively (Fig. 8B). In Arabidopsis, the concentration of linalyl-β-d-glucoside was under the limit of detection in the WT control. However, transgenic plants (T4163, T4276, and T4246) produced readily detectable linalyl-β-d-glucoside (Fig. 8C). These results showed that *PpUGT85A2* synthesizes linalyl-β-d-glucoside formation *in planta*.

In peach fruit, levels of other glycosylated volatiles did not change significantly after infiltration with *PpUGT85A2* (Fig. 8D). This may be related to the content of free volatiles as substrates. The contents of volatiles such as 2-phenylethanol, benzyl alcohol, geraniol, and eugenol were under the detection limits in peach fruit (Supplementary Table S4; Supplementary Fig. S10D). In tobacco and Arabidopsis, the presence of free volatiles as potential substrates for the enzyme was detected (Supplementary Fig. S10E, F). A significant increase in the glycosylated form of 2-phenylethanol was observed in transgenic plants overexpressing peach *PpUGT85A2* (Fig. 8E, F), and similar accumulation was also observed for geraniol in tobacco.

### Subcellular localization of PpUGT85A2

**Fig. 9.**
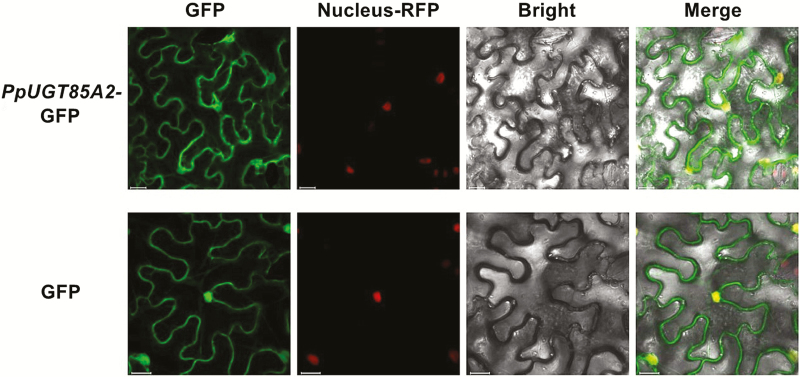
Subcellular localization of PpUGT85A2 in *Nicotiana benthamiana* leaves. GFP, GFP channel; Nucleus–RFP, transgenic tobacco plants with red fluorescence in the nucleus; Bright, light microscopy image; Merge, merged image of the GFP and Bright channels. Scale bars represent 20 μm.

To determine the subcellular location of PpUGT85A2, a recombinant PpUGT85A2 tagged at the C-terminus with GFP was constructed, and the vector *35S::PpUGT85A2*-GFP was infiltrated into tobacco leaves. The green fluorescence signal of the fusion protein indicated that GFP-tagged PpUGT85A2 was located in both the cytoplasm and nucleus (Fig. 9). Three independent experiments showed the same subcellular localization of PpUGT85A2. A similar pattern of localization has been observed for other UGTs in plants, including zeatin *O*-glucosyltransferase UGT85A1 (Jin *et al.*, 2013), UGT87A2 involved in flower development regulation (Wang *et al.*, 2012), and UGT73C6. This latter UGT glycosylates flavonoids (Jones *et al.*, 2003), hydroxycoumarins (Lim *et al.*, 2003), and brassinosteroids (Husar *et al.*, 2011). UGT enzymes may function in the nucleus to protect nuclear components from toxins (Hart *et al.*, 1989).

## Discussion

Glycosylated bound volatiles are potentially important sources of aroma formation. A substantial portion of potential aroma volatiles can exist as glycosides. Understanding and ultimately limiting conjugation has the potential to impact fruit flavor positively. Biosynthesis of these non-volatile compounds is catalyzed by UGT enzymes that attach sugars to volatile compounds (Schwab *et al.*, 2015*a*). One important peach flavor volatile where a significant portion of the total pool is present as glycoside is linalool. Glycosylated linalool plays an important role to flavor and consumer perception by retronasal perception (Parker *et al.* 2017), but the *UGT* gene(s) associated with glycosylation have not been identified in peach. Here, we identified a gene, *PpUGT85A2*, encoding an enzyme with linalool glycosylation activity by exploiting the correlation between the metabolome and transcriptome across different tissues, fruit developmental stages, treatments, and peach cultivars. Since there have been no successful reports of stable transgenic peach fruits, transient overexpression experiments in peach fruit and stable overexpression experiments in transgenic tobacco and Arabidopsis were conducted to validate the function of *PpUGT85A2* in production of linalyl-β-d-glucoside *in vivo*. We demonstrated that *PpUGT85A2* is associated with linalool glycosylation based on evidence from both *in vitro* assay of recombinant protein and *in vivo* transgene expression.

The diverse functions of linalool, ranging from pollinator attraction to toxin, were reviewed by Raguso (2016), where these two themes are suggested to be equally relevant to the ecology and evolution of volatiles. Free linalool directly contributes to a sweet, floral and alcoholic note, and is associated with consumers’ liking of peach fruit (Liu *et al.*, 2017; Tian *et al.*, 2017). As a major component of aroma and flavor, free linalool was also detected in fruits and flowers of many species (Lewinsohn *et al.*, 2001; Schwab *et al.*, 2015*a*; Li *et al.*, 2017). However, the available evidence suggests that linalool is toxic to plant cells at high dosages. Passing through long-term evolution, fruits must have developed mechanisms that regulate the levels of free linalool, producing a fruit that is attractive to seed-dispersing animals while limiting potential toxic effects of free linalool. Conversion of linalool to non-volatile conjugates is likely to have evolved in plants as a strategy for detoxification (Raguso and Pichersky, 1999). Previous attempts to overproduce volatile *S*-linalool by overexpressing linalool synthase genes in *Petunia hybrida* (Lücker *et al.*, 2001) and Arabidopsis (Aharoni *et al.*, 2003) often resulted in the accumulation of linalyl-β-d-glucoside. In the present study, feeding with extra free linalool caused a significant increase in production of glycosylated linalool, indicating that linalyl-β-d-glucoside formation is induced by linalool. An increased concentration of linalyl-β-d-glucoside was observed in ripe peach fruit, along with rapid accumulation of free linalool. A positive correlation was also observed between linalool and the glucoside in peach fruit cultivars whose *PpUGT85A2* expression varied by >30-fold. The data strongly suggest that *PpUGT85A2* expression is regulated by linalool content and is directly responsible for conversion of linalool to its glucoside.

Non-volatile linalyl-β-d-glucoside acted as a potential sensory attribute to enhance flavor quality during tasting in the mouth via enzymes in saliva (Parker *et al.* 2017). As a hidden aroma source, glycosylated linalool and monoterpenes could be released into free forms during fruit wine fermentation, therefore affecting flavor quality (Li *et al.*, 2017). Linalyl-β-d-glucoside was widely detected in fruit such as grape (Bönisch *et al.*, 2014), kiwifruit (Yauk *et al.*, 2014), mandarin (Fan *et al.*, 2009), and peach (Wu *et al.*, 2017). Although PpUGT85A2 showed lower enzymatic activity towards linalool than other substrates *in vitro*, similar results were also observed for other plant UGTs. Grape UGT14a showed ~20% activity toward linalool relative to geraniol; no linalool activity was observed for UGT15 and UGT16 (Bönisch *et al.*, 2014). A low activity of UGT leaving some unconjugated linalool might be favorable for fruit flavor quality. There may exist other UGTs that could convert free linalool to the glycosylated form; however, PpUGT85A2 had the highest enzyme activity toward free linalool among 12 peach UGTs tested in the present study. Production of terpene glucosides catalyzed by UGTs has commercial applications for fruit, food, cosmetic, and pharmaceutical industries due to slow release of linalool (Schwab *et al.*, 2015a, b).

Evidence from transgenic sweet orange showed that linalool may act as both a direct antibacterial agent and a signal molecule involved in triggering a non-host resistance response (Shimada *et al.*, 2017). Glycosylated linalool is also a kind of storage form and potential release source for defense or signaling in plants (Schwab *et al.*, 2015*b*). After treatment with SA, peach fruit produced significantly more linalool, both the free and glycosylated forms. Therefore, linalool was suggested to be a mobile info-compound for the activation of defense response in plants (Taniguchi *et al.*, 2014). Based on these results, formation of linalyl-β-d-glucoside may be associated with defense response in peach fruit after SA treatment.

Glycosylated linalool is the most abundant monoterpene conjugate in peach fruit, and is physiologically important for flavor, for defense, for fruit consumption, and for seed dispersal. We demonstrated that PpUGT85A2 catalyzes the glycosylation of linalool, whose transcript levels are induced by ethylene and tend to accumulate as peach fruit ripen. Our findings provided molecular insights into regulation of volatile contents in peach fruit. The biosynthesis of glycosylated linalool catalyzed by UGTs could translate into molecular tools to regulate fruit flavor quality and to understand the defense system during plant evolution.

## Supplementary data

Supplementary data are available at *JXB* online.

**Table S1.** Correlation between transcript levels of peach *UGT* genes and linalyl-β-d-glucoside during fruit development.

**Table S2.** Correlation between transcript levels of peach *UGT* genes and linalyl-β-d-glucoside in response to ethylene and 1-MCP treatment.

**Table S3.** The kinetic parameters analysis of PpUGT85A2.

**Table S4.** Concentration of volatiles in peach fruit during development and ripening.

**Table S5.** Primer sequences used in the present study.

**Fig. S1.** Content of free linalool in peach fruit after ethylene and 1-MCP treatment.

**Fig. S2.** Transcript levels of 20 peach *UGT* genes in peach fruit after ethylene and 1-MCP treatment.

**Fig. S3.** Effect of SA treatment on content of free linalool in peach fruit.

**Fig. S4.** Transcript levels of 20 peach *UGT* genes in peach fruit after UV-B irradiation.

**Fig. S5.** SDS–PAGE analysis of PpUGT85A2 protein.

**Fig. S6.** Enzymatic activity of PpUGT85A2 towards different sugar donors.

**Fig. S7.** Relative enzymatic activity of PpUGT85A2 protein toward different putative substrates.

**Fig. S8.** Chiral GC-MS analysis of linalool enantiomers in peach fruit.

**Fig. S9.** Relative expression of *PpUGT85A2* in transiently overexpressing peach fruits.

**Fig. S10.** Changes in free volatiles in plants overexpressing peach *PpUGT85A2*.

Supplementary Material
