# Supplementary material for: UDP-glucosyltransferase PpUGT85A2 controls volatile glycosylation in peach
Source: J Exp Bot. 2018 Nov 27;70(3):925–36. doi: 10.1093/jxb/ery419 (PMC6363097; doi:10.1093/jxb/ery419)
Supplement: Supplementary Material [file ery419_suppl_supplementary_material.pdf]

Supplementary Data

Supplementary Table S1. Correlation between transcript levels of peach *UGT* genes and linalyl-β-D-glucoside during fruit development. # represented 12 *UGT* genes cloned for enzymatic activity analysis.

	correlation	p-value
Prupe.1G519600#	0.95111	5.28E-08
Prupe.7G124200#	0.87066	2.41E-05
Prupe.1G547800	0.86834	2.69E-05
Prupe.1G169300	0.84873	6.31E-05
Prupe.8G130200	0.82579	0.000149
Prupe.7G149200	0.81125	0.000242
Prupe.4G117900#	0.80443	0.000299
Prupe.3G166800	0.79087	0.000447
Prupe.1G169100	0.64328	0.009677
Prupe.3G190100#	0.52691	0.043575
Prupe.6G189900#	-0.32656	0.23485
Prupe.1G053300#	-0.32781	0.23296
Prupe.6G190100#	-0.3834	0.15834
Prupe.6G008000#	-0.43013	0.10952
Prupe.6G008300#	-0.54688	0.034888
Prupe.1G520000#	-0.59936	0.018207
Prupe.8G063500#	-0.60985	0.015781
Prupe.6G189800#	-0.67572	0.005692
Prupe.1G505100	-0.6888	0.004514
Prupe.1G552100	-0.83743	9.8E-05

Supplementary Table S2. Correlation between transcript levels of peach *UGT* genes and linalyl-β-D-glucoside in response to ethylene and 1-MCP treatment. # represented 12 *UGT* genes cloned for enzymatic activity analysis.

	correlation	p-value
Prupe.1G519600#	0.94055	0.000159
Prupe.3G190100#	0.7514	0.019584
Prupe.1G547800	0.72574	0.02687
Prupe.6G189900#	0.65567	0.055191
Prupe.1G552100	0.30821	0.41972
Prupe.7G149200	0.26996	0.48235
Prupe.8G130200	0.2069	0.59326
Prupe.1G169300	-0.09744	0.80307
Prupe.3G166800	-0.12003	0.7584
Prupe.1G505100	-0.27496	0.47396
Prupe.1G053300#	-0.40446	0.28027
Prupe.6G190100#	-0.40446	0.28027
Prupe.6G008300#	-0.43072	0.24713
Prupe.4G117900#	-0.53799	0.13515
Prupe.1G520000#	-0.61743	0.076457
Prupe.1G169100	-0.67131	0.047721
Prupe.8G063500#	-0.79435	0.010552
Prupe.7G124200#	-0.80196	0.009322
Prupe.6G008000#	-0.83641	0.004949
Prupe.6G189800#	-0.87253	0.002145

Supplementary Table S3. The kinetic parameters analysis of PpUGT85A2.

Substrate	K_m (mM)	k_{cat} (s^{-1})	k_{cat} / K_m (s^{-1} M^{-1})
Eugenol	0.262±0.157	0.996±0.190	3802
2-Phenylethanol	0.448±0.110	1.613±0.454	3600
Benzyl alcohol	0.236±0.040	0.115±0.015	487
Geraniol	4.216±1.647	0.109±0.025	26

Supplementary Table S4. Concentration of volatiles in peach fruit during development and ripening.

Compound	Type	Days after bloom (DAB)				
		34	71	94	108	111
α-Terpineol	Free	UD	UD	4.21±4.21	12.04±1.24	20.46±1.45
	β-D-Glucosides	UD	UD	UD	7.91±0.74	14.78±2.76
Geraniol	Free	UD	UD	UD	UD	UD
	β-D-Glucosides	109.10±31.89	19.89±1.19	11.13±4.00	8.19±0.65	11.05±2.92
Benzyl Alcohol	Free	UD	UD	UD	UD	UD
	β-D-Glucosides	115.43±23.02	238.71±50.39	356.40±69.33	135.05±18.91	236.08±16.06
2-Phenylethanol	Free	UD	UD	UD	UD	UD
	β-D-Glucosides	118.09±23.87	81.23±12.80	55.63±15.26	37.25±5.04	55.17±9.39
Eugenol	Free	UD	UD	UD	UD	UD
	β-D-Glucosides	126.91±39.71	315.18±28.28	631.57±201.16	307.81±46.34	369.44±75.64

UD, under the limit of detection.

Supplementary Table S5. Primer sequences used in the present study.

Primers	Sequence (5'→ 3')	Description
qPCR-PpUGT85A2-FP1	CACTCAGCCATTGGAGGGTT	RT-qPCR of *PpUGT85A2*
qPCR-PpUGT85A2-RP1	CAGCAGATGAGAGGCACTCC	RT-qPCR of *PpUGT85A2*
qPCR-PpUGT85A2-FP2	AGCAAAGTGCTTCTTTCCCCA	RT-qPCR of *PpUGT85A2*
qPCR-PpUGT85A2-RP2	AAAAGCCTCGGCAAACGGTA	RT-qPCR of *PpUGT85A2*
pET-PpUGT85A2-FP	AAGGCCTCTGTCGACATGAGTCCAGTTGCCTCCAAAG	pET vector cloning of *PpUGT85A2*
pET-PpUGT85A2-RP	AGAATTCGCAAGCTTCTAATCTCTTGGGGAAAGAAGC	pET vector cloning of *PpUGT85A2*
SK-PpUGT85A2-FP	GCCCAAGCTGAGCTCATGAGTCCAGTTGCCTCCAAAG	pGreen-SK vector cloning of *PpUGT85A2*
SK-PpUGT85A2-RP	GACTCTAGAGGATCCATCTCTTGGGGAAAGAAGCACT	pGreen-SK vector cloning of *PpUGT85A2*
GFP-PpUGT85A2-FP	CTCGGTACCATGAGTCCAGTTGCCTCCAAAG	35S-eGFP vector cloning of *PpUGT85A2*
GFP-PpUGT85A2-RP	CATGTCGACATCTCTTGGGGAAAGAAGCACT	35S-eGFP vector cloning of *PpUGT85A2*
PpTEF2-FP	GGTGTGACGATGAAGAGTGATG	RT-qPCR of house-keeping gene *PpTEF*
PpTEF2-RP	TGAAGGAGAGGGAAGGTGAAAG	RT-qPCR of house-keeping gene *PpTEF*

The restriction enzyme sites are labeled.

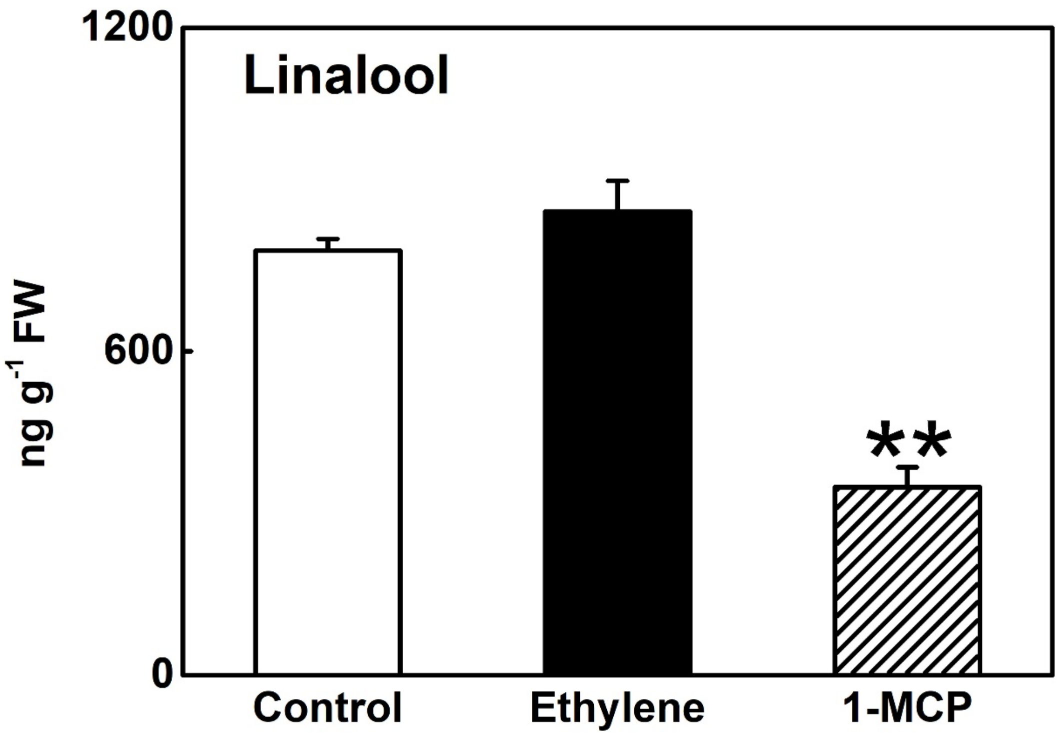

Supplementary Fig. S1. Content of free linalool in peach fruit after ethylene and 1-MCP treatment. Data are presented as mean $\pm$ standard error from three independent biological replicates. Significant differences are compared against control and indicated with asterisks above the bars (** $P < 0.01$).

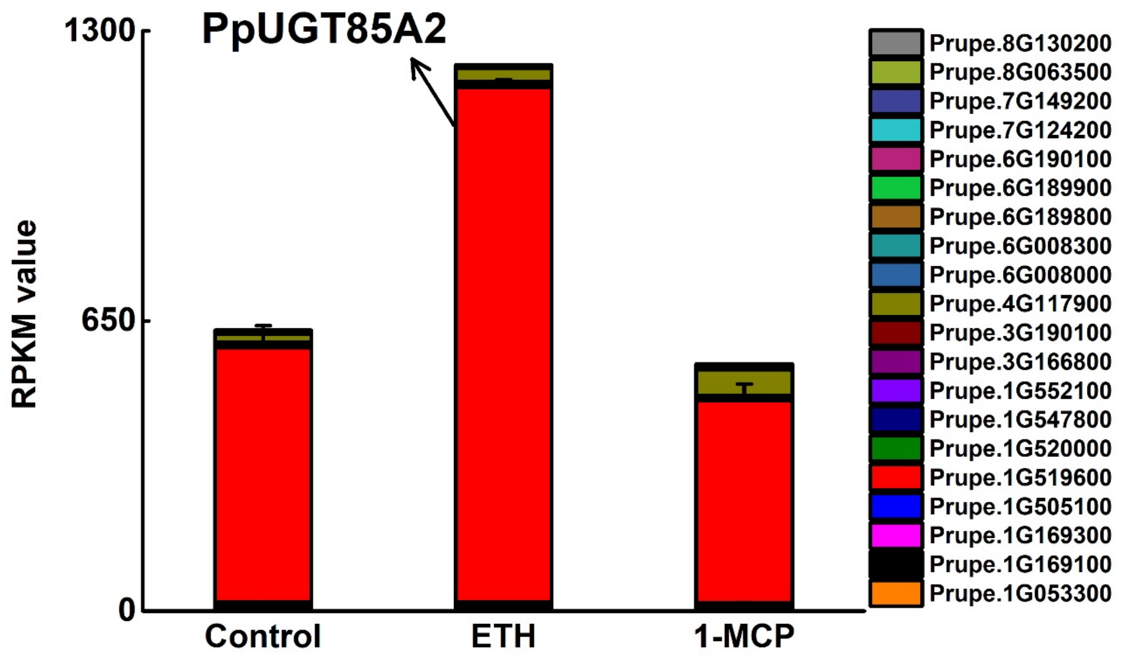

Supplementary Fig. S2. Transcript levels of 20 peach *UGT* genes in peach fruit after ethylene and 1-MCP treatment.

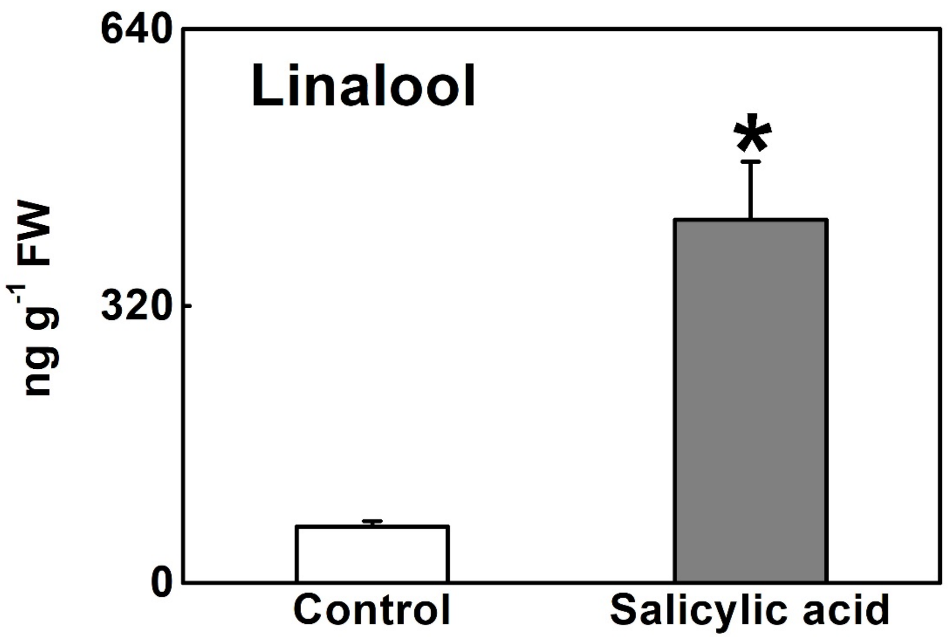

Supplementary Fig. S3. Effect of SA treatment on content of free linalool in peach fruit. Data are presented as mean ± standard error from three independent biological replicates. Significant differences are indicated with asterisks above the bars (**P* < 0.05).

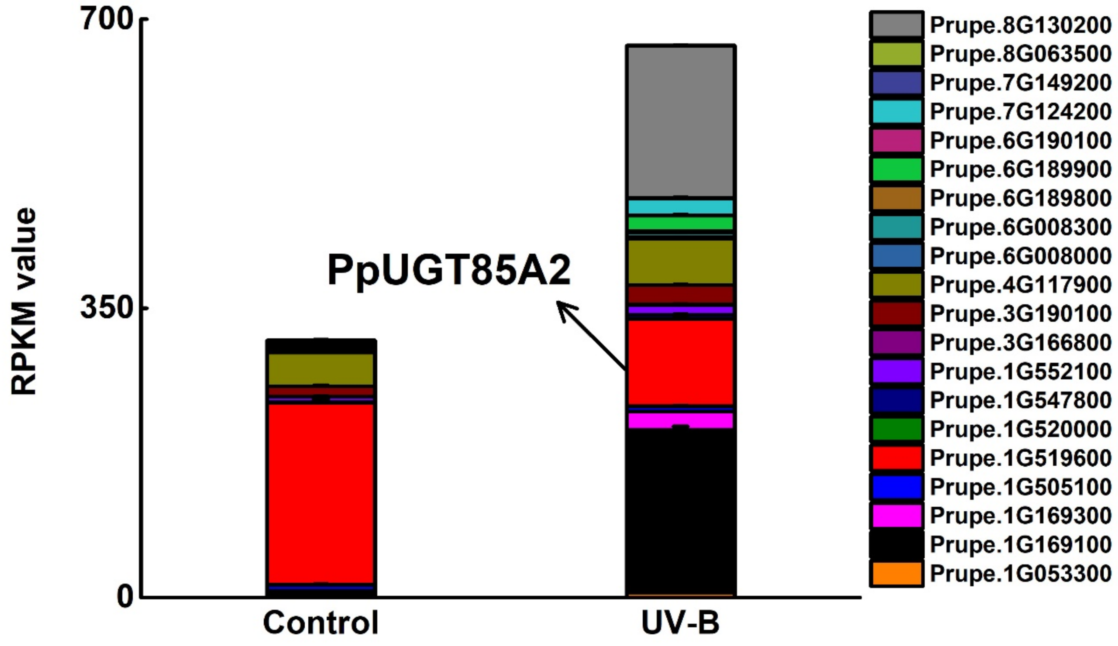

Supplementary Fig. S4. Transcript levels of 20 peach *UGT* genes in peach fruit after UV-B irradiation.

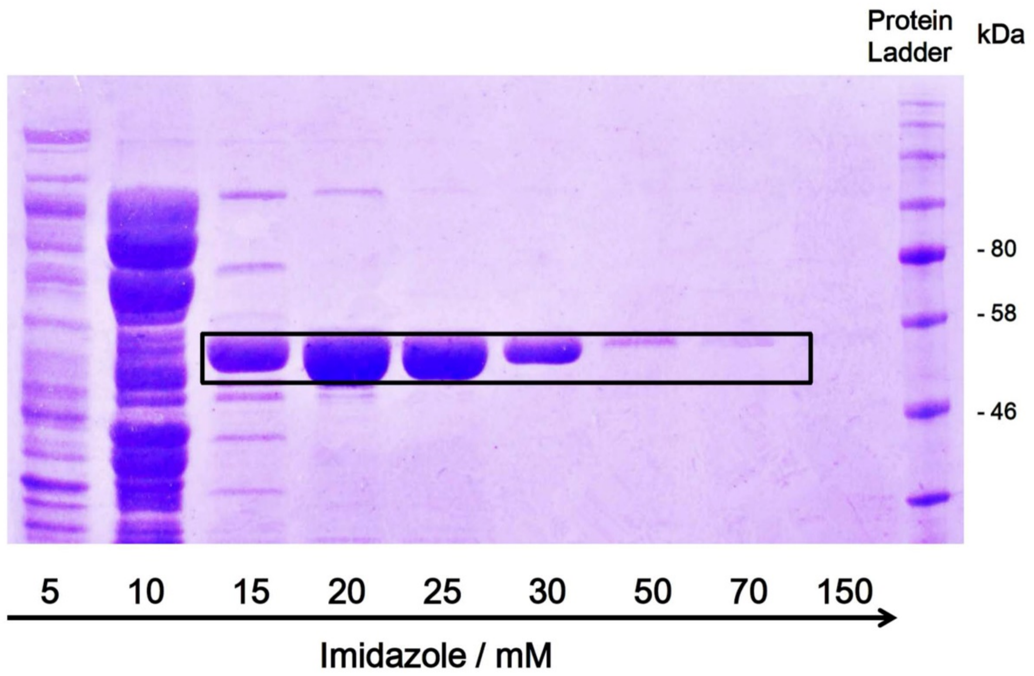

Supplementary Fig. S5. SDS-PAGE analysis of PpUGT85A2 protein.

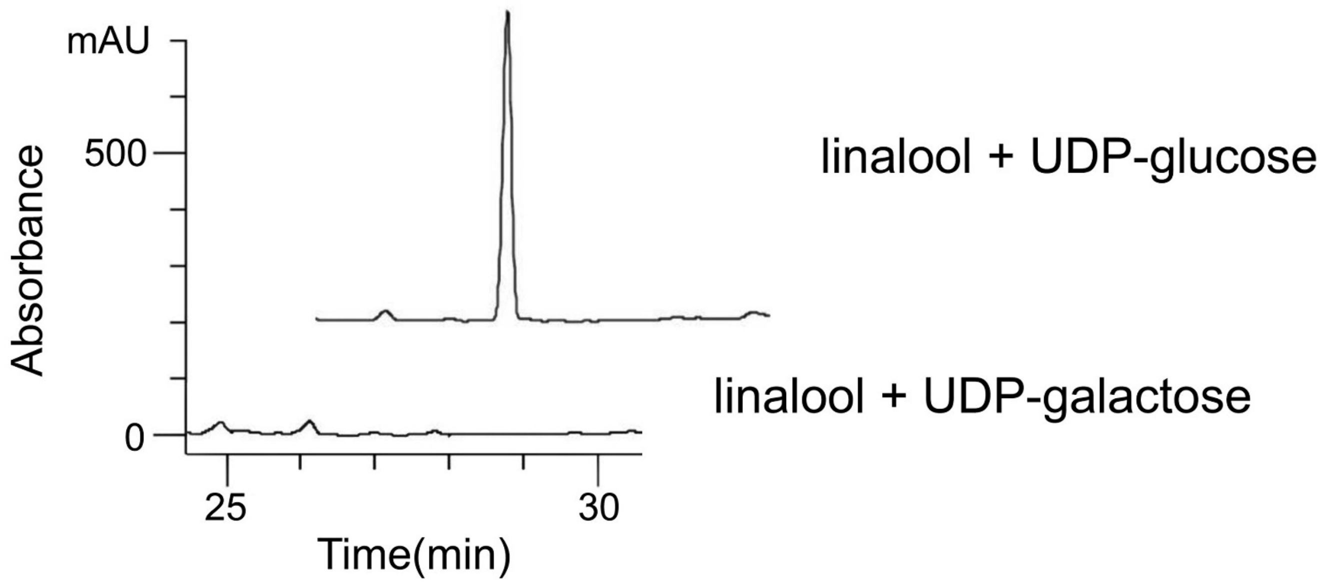

Supplementary Fig. S6. Enzymatic activity of PpUGT85A2 towards different sugar donors.

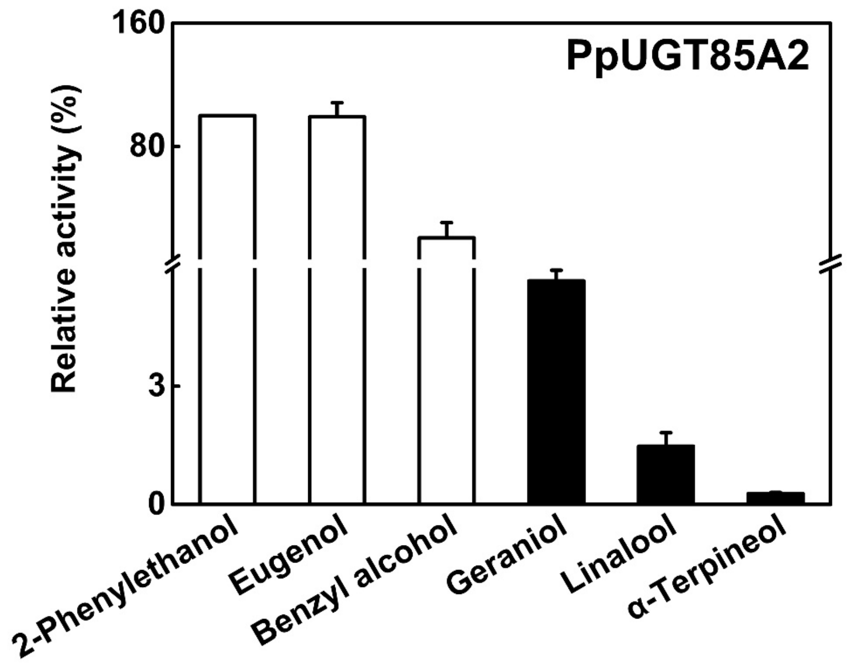

Supplementary Fig. S7. Relative enzymatic activity of PpUGT85A2 protein toward putative different substrates. UGT activity towards 2-phenylethanol is set at 100%. Data are presented as mean ± standard error from three independent biological replicates.

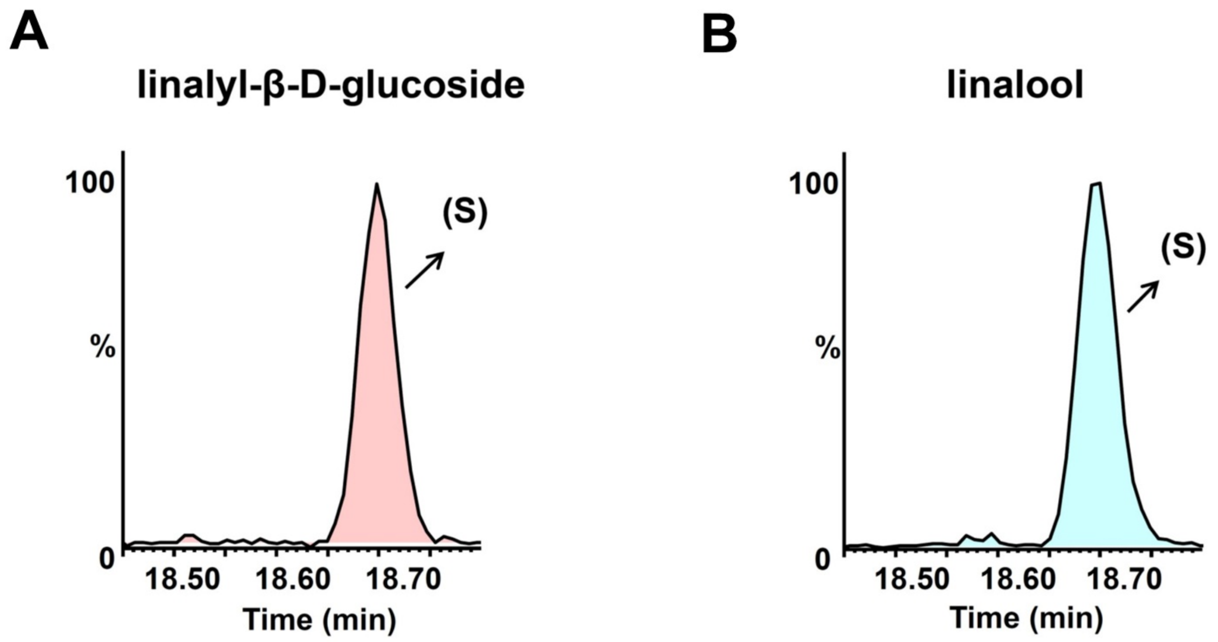

Supplementary Fig. S8. Chiral GC-MS analysis of linalool enantiomers in peach fruit. Linalyl-β-D-glucoside (A) and free linalool (B) in peach fruit.

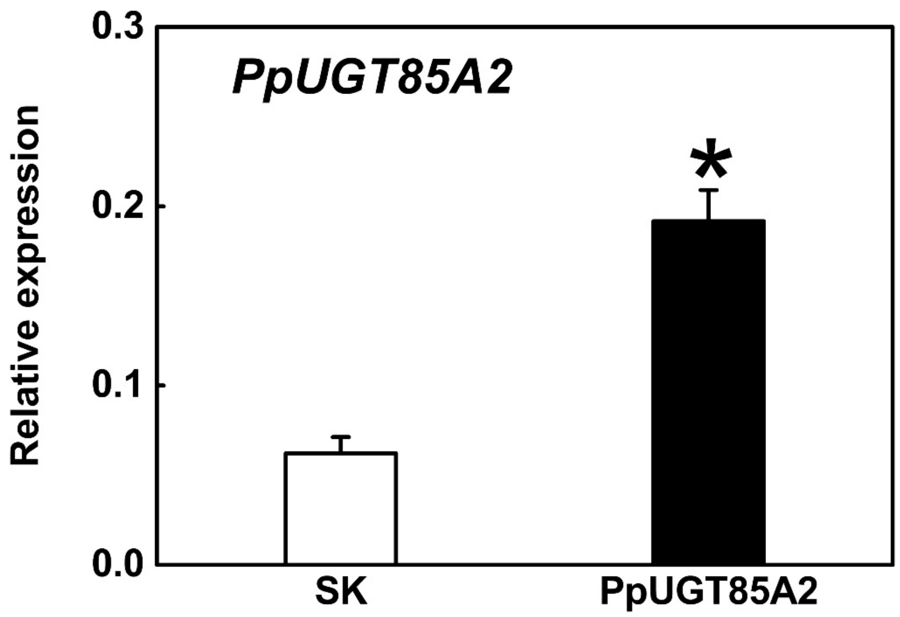

Supplementary Fig. S9. Relative expression of *PpUGT85A2* in transiently over-expressed peach fruits. Relative expression levels were determined using qPCR. Empty SK vector was used as a control. Data are presented as mean ± standard error from three independent biological replicates. Significant differences are indicated with asterisks above the bars (* $P < 0.05$).

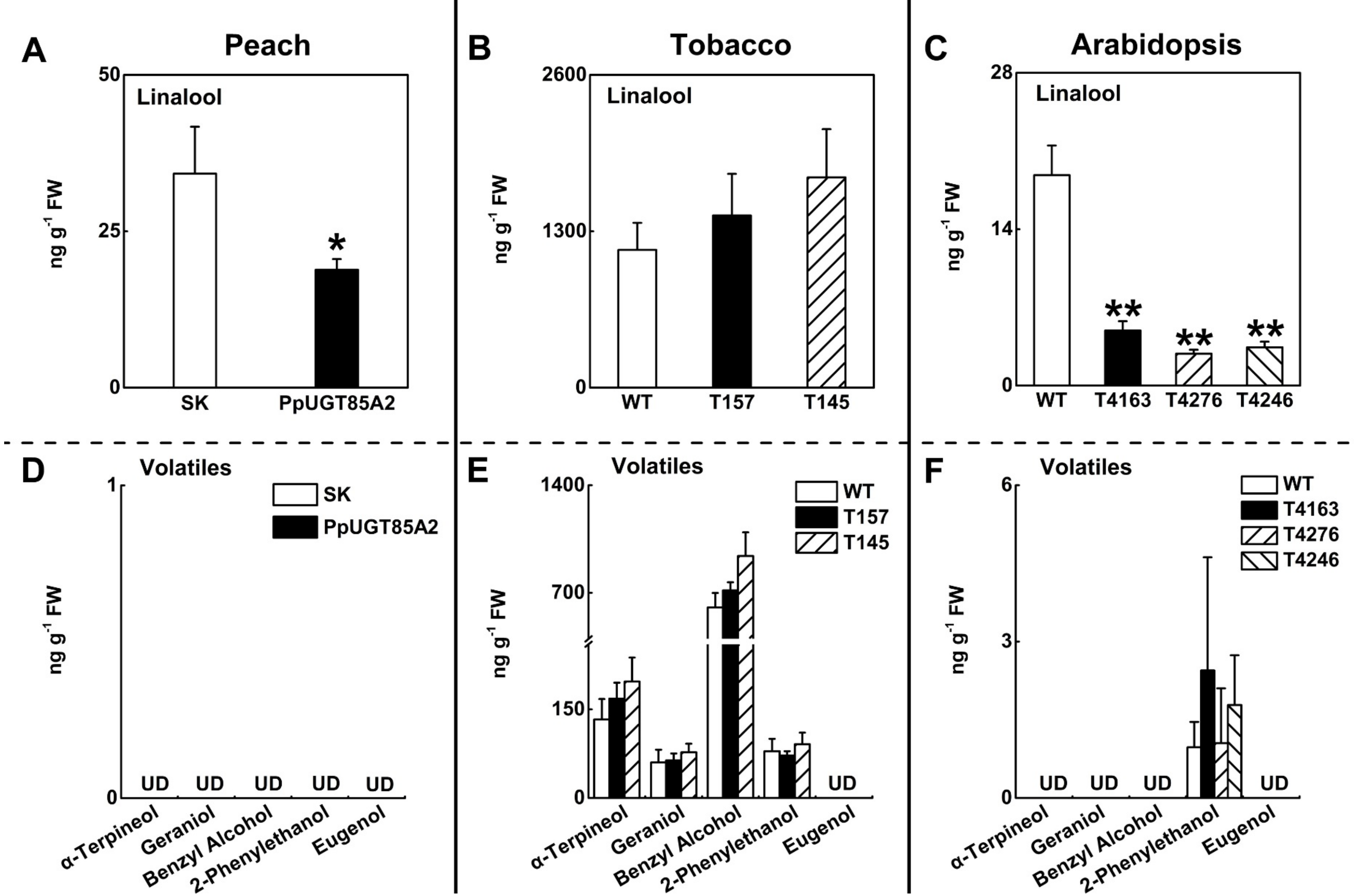

Supplementary Fig. S10. Changes in free volatiles in plants over-expressing peach *PpUGT85A2*. Production of linalool and other volatiles were produced by peach fruit (A, D), by tobacco (B, E), and Arabidopsis plants (C, F). Peach fruit were transiently overexpressed PpUGT85A2. Empty SK vector was used as a control. For tobacco and Arabidopsis transgenic plants, wild-type (WT) plants were used as controls. Data are presented as mean ± SE from three independent biological replicates. Significant differences are compared against empty SK vector or wild-type (WT), and indicated with asterisks above the bars (* $P < 0.05$, ** $P < 0.01$). UD, under the limit of detection.
